# Conductive Supramolecular Polymer Nanocomposites with Tunable Properties to Manipulate Cell Growth and Functions

**DOI:** 10.3390/ijms23084332

**Published:** 2022-04-14

**Authors:** Cheng-You Wu, Ashenafi Zeleke Melaku, Fasih Bintang Ilhami, Chih-Wei Chiu, Chih-Chia Cheng

**Affiliations:** 1 Graduate Institute of Applied Science and Technology, National Taiwan University of Science and Technology, Taipei 10607, Taiwan; johnnywu2262756@gmail.com (C.-Y.W.); ashenafichem@gmail.com (A.Z.M.); fasihilhami17@gmail.com (F.B.I.); 2 Department of Materials Science and Engineering, National Taiwan University of Science and Technology, Taipei 10607, Taiwan; cwchiu@mail.ntust.edu.tw; 3 Advanced Membrane Materials Research Center, National Taiwan University of Science and Technology, Taipei 10607, Taiwan

**Keywords:** bioactive supramolecular polymer, conductive graphene nanosheet, cell culture, hydrogen bonding, indirect electrical stimulation

## Abstract

Synthetic bioactive nanocomposites show great promise in biomedicine for use in tissue growth, wound healing and the potential for bioengineered skin substitutes. Hydrogen-bonded supramolecular polymers (3A-PCL) can be combined with graphite crystals to form graphite/3A-PCL composites with tunable physical properties. When used as a bioactive substrate for cell culture, graphite/3A-PCL composites have an extremely low cytotoxic activity on normal cells and a high structural stability in a medium with red blood cells. A series of in vitro studies demonstrated that the resulting composite substrates can efficiently interact with cell surfaces to promote the adhesion, migration, and proliferation of adherent cells, as well as rapid wound healing ability at the damaged cellular surface. Importantly, placing these substrates under an indirect current electric field at only 0.1 V leads to a marked acceleration in cell growth, a significant increase in total cell numbers, and a remarkable alteration in cell morphology. These results reveal a newly created system with great potential to provide an efficient route for the development of multifunctional bioactive substrates with unique electro-responsiveness to manipulate cell growth and functions.

## 1. Introduction

Polymer-based bioengineering approaches have risen to prominence in the biomedical field as potential materials for use in fabrication with a wide range of tissue engineering applications [1,2,3,4]. Several studies have shown that synthetic bioactive polymers with a tailorable structural composition, surface microstructure and wettability can substantially affect cellular response and growth through the addition of specific functional monomers and control of the monomer addition sequence during the polymerization process [5,6,7]. However, the development of synthetic bioactive polymers is frequently constrained by our limited understanding of how cell adhesion and proliferation are regulated by the extracellular matrix (ECM) [8,9,10,11]. Recently, supramolecular polymers produced via reversible non-covalent interactions have attracted attention owing to the unique mechanical properties of their matrices, such as their environmental stimuli-responsiveness [12,13], self-healing [14,15] and shape-memory behavior [16,17]. Specifically, supramolecular hydrogen-bonding moieties (or synthons) within the polymer that drive self-assembly behavior might be exploited to manipulate supramolecular polymer–cell junctions [18,19,20,21,22]. For example, Dankers and coworkers synthesized novel synthetic supramolecular polymers with quadruple hydrogen-bonding ureido-pyrimidinone (UPy) moieties that could spontaneously self-assemble into a membrane-like structure and improve the bioactivity of cell growth and proliferation, thereby achieving a biocompatible polymer [23,24]. Therefore, functional polymeric materials with supramolecular moieties and strong hydrogen-bonding capability have critical factors required for the development of multifunctional tissue engineering scaffolds with tunable physical properties that can enhance the overall growth of cultured cells.

Graphene is a one-atom-thick two-dimensional material with a hexagonal structure that provides distinct features, including a large surface area, excellent thermo-electrical conductivity, high mechanical strength and light transmittance [25,26,27]. In particular, conductive materials such as graphene have the potential to provide an essential role in tissue regeneration that can accelerate cell adhesion and migration during wound healing by electrical stimulation. Cells have an innate self-electroactivity ability that enhances the overall efficiency of their cellular wound healing after electrical stimulation [28,29,30,31,32]. Nevertheless, full carbon-based graphene nanosheets have limitations. In particular, they are extremely hydrophobic, making it a challenge to interface them with biological systems and thus causing the significant inhibition of cell growth and functions [28,29,30]. Given the hydrophobic nature of graphene, we speculate that introducing hydrogen-bonded supramolecular polymers into the graphene matrix could significantly improve the hydrophilicity of the composites, thereby improving the affinity between the composite substrate and cells [18,19]. We further propose that electrical stimulation—as an exogenous physiological stimulus—could enhance the cellular affinity of adhesion, proliferation, and differentiation on materials substrates [33,34,35]. As a promising strategy with electrical stimuli, direct electrical stimulation (DES) implies the interaction of electron transport chain components with a working electrode surface into cells which may cause burn damage to living tissues treated with DES [36]. In contrast, indirect electrical stimulation (IES) involves the transfer of electrons from a working electrode to a microorganism without direct interaction into the cellular environment that allows safe stimulation to promote cellular growth [37]. Therefore, we speculated that a combination of hydrogen-bonded supramolecular polymers with graphene nanosheets for cell and tissue culture using IES may old great potential as a high-performance conductive bioactive substrate for manipulating cells in engineered tissues.

**Scheme 1:**
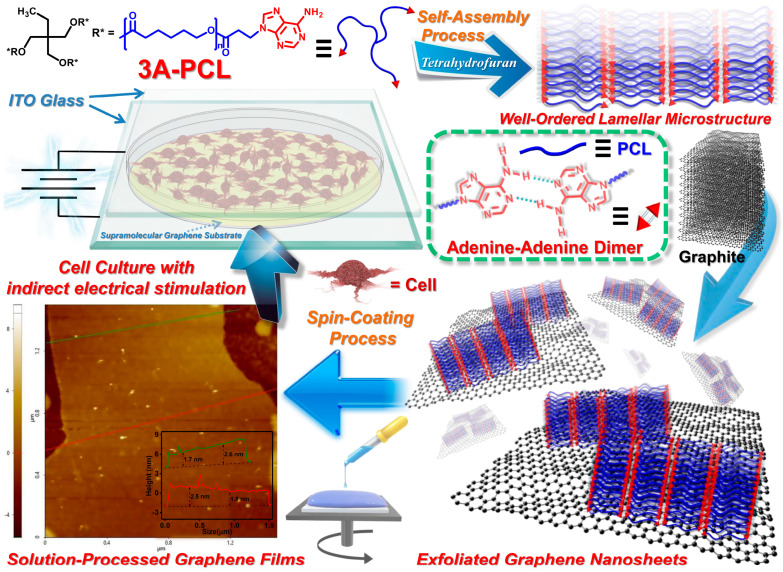
Graphical illustration for the development of supramolecular composites: self-assembled lamellar nanostructures are formed by hydrogen-bonded adenine units within the 3A-PCL macromer, which subsequently adsorbs on the surface of exfoliated graphene nanosheets after blending with graphite in THF. The resulting composite is applicable as a conductive bioactive substrate for cell culture under indirect electrical stimulus.

Recent studies in our laboratory demonstrated that supramolecular exfoliated graphene nanosheets with tunable physical properties could be obtained by controlling the amount of three-arm adenine-end-capped polycaprolactone polymer (3A-PCL) [38]. This results from the strong affinity of the self-assembled lamellar and spherical nanostructures of 3A-PCL to be strongly absorbed onto the surface of the graphite crystals, which subsequently lead to the formation of exfoliated graphene nanosheets. The tailorable graphene-exfoliation level and controlled conductive performance of these graphite/3A-PCL composites inspired us to explore their ability as a conductive bioactive substrate for cell culture in vitro (Scheme 1).

The objectives of this work were to achieve improved the surface bioactivity of conductive graphite/3A-PCL substrates to promote the adhesion, migration and proliferation of the cells cultured on the substrates via IES at very low voltage levels. In this paper, we show that graphite/3A-PCL composites not only exhibit extremely low cytotoxic activity against normal cells and high structural stability in a red blood cell-containing medium, but also significantly enhance wound-healing and cell-growth rates. In addition, we showed that graphite/3A-PCL substrates under an indirect current electric field at only 0.1 V can rapidly and efficiently produce cell adhesion, spreading and proliferation, resulting in a substantial increase in the total cell numbers and significant alteration in cell morphology. To the best of our knowledge, this is the first study demonstrating a conductive bioactive supramolecular substrate based on hydrogen-bonded adenine units and exfoliated graphene nanosheets that can efficiently control cell growth when exposed to IES. This newly created system has an advantageous combination of composite, amphipathic, conductive and bioactive characteristics with promise as a multifunctional, soft cell-culture scaffold for skin substitute bioconstructs and tissue-engineered regeneration.

## 2. Results and Discussion

**Figure 1:**
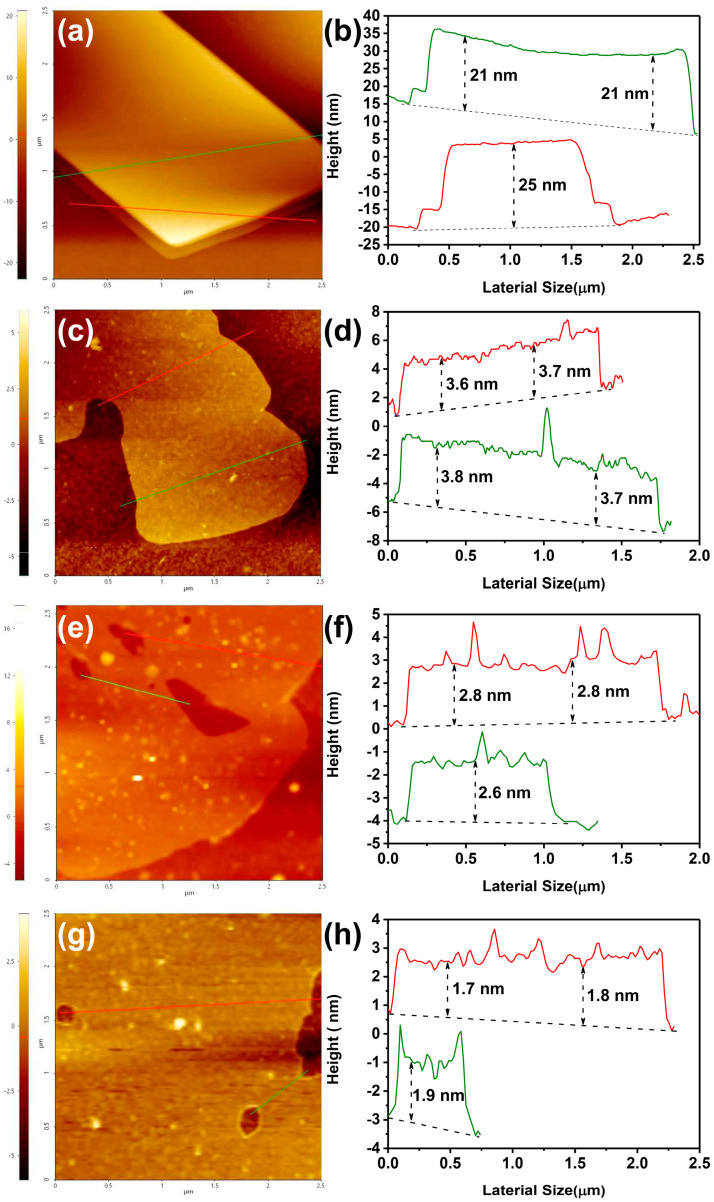
Atomic force microscopy (AFM) images (left side) and corresponding height profiles (right sides) of (**a**,**b**) pristine crystal graphite and the resulting graphite/3A-PCL composites at (**c**,**d**) 5/10, (**e**,**f**) 3/10 and (**g**,**h**) 1/10 blend ratios. The green and red profiles presented in (**b**,**d**,**f**) and (**h**) correspond with the green and red solid lines seen in the images of (**a**,**c**,**e**,**g**), respectively.

The molecular structure of tri-adenine end-capped polycaprolactone macromer (3A-PCL) and its direct self-assembly into well-ordered lamellar structures in solid state via the noncovalent linkage of the self-complementary adenine-adenine (A-A) hydrogen-bonding interactions are shown in the upper part of Scheme 1. When 3A-PCL is directly blended with natural graphite in tetrahydrofuran (THF) using ultrasonic treatment, 3A-PCL efficiently undergoes self-assembly in THF to form a stable lamellar structure on the graphite surface. This promotes the exfoliation of the bulk graphite into variable layers of graphene nanosheets. The number of layers depends on the amount of 3A-PCL incorporated into the graphite crystals (Figure 1) due to the presence of the strong noncovalent intermolecular interactions between the exfoliated graphene surface and the self-organized lamellar 3A-PCL nanostructures. A well-tunable layer number and physical properties of the graphite/3A-PCL nanosheets in the solid state were analyzed and discussed in detail in our previous work [38]. Here, we expanded on our previous work, exploring the physical properties and potential applications for the cell culturing of graphite/3A-PCL composites.

### 2.1. Physical Properties of Graphite/3A-PCL Composites

**Figure 2:**
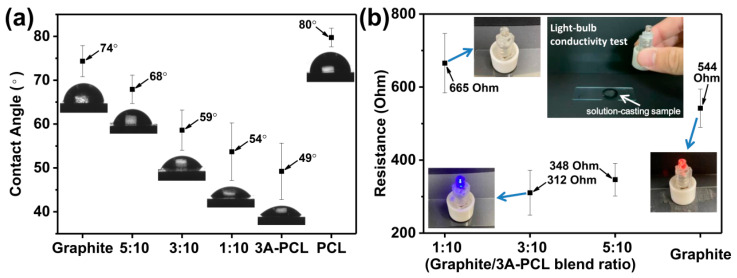
(**a**) WCA analysis of pristine graphite, graphite/3A-PCL composites, 3A-PCL and commercial PCL; (**b**) electrical resistance values for the graphite and graphite/3A-PCL composites. The photographs in the inset of (**b**) present the conductivity performance of graphite and graphite/3A-PCL composites through a simple light bulb conductivity apparatus.

To further extend our previous findings, we explore here the effects of the self-assembled lamellar structures on the surface wettability of graphite/3A-PCL composites at 25 °C by measuring the water contact angle (WCA). Spin-coated commercial polycaprolactone (PCL; average molecular weight = 80,000 g/mol) and adenine-functionalized 3A-PCL thin-films had WCA values of approximately 80° and 49°, respectively. This confirms that introducing adenine moieties into the end groups of the PCL oligomer increases the surface hydrophilicity of 3A-PCL (Figure 2a). The WCA values of all spin-coated graphite/3A-PCL thin-films exhibited a gradual decrease from 74° to 54° with increasing weight fractions of 3A-PCL content, indicating that adjusting the content of 3A-PCL within composites not only significantly affected the surface hydrophilicity of composites, but also effectively regulated their level of surface wettability.

An ideal exfoliated graphene-based composite for engineering applications must have high electrical conductivity in a thin-film state. We investigated the electrical resistance of spin-coated graphite/3A-PCL films using a light bulb conductivity apparatus at 25 °C. The light bulbs instantly lit up after being placed on the substrates of the 3/10 and 5/10 graphite/3A-PCL composites, but not on the 1/10 graphite/3A-PCL composite (Figure 2b). This suggests that an increased proportion of graphite forms enough exfoliated graphene nanosheets to enable overall electrical conductivity in composites. In addition, a four-point probing measurement of sensor resistance at 25 °C and relative humidity of approximately 35% showed similar trends for all composites (Figure 2b), further demonstrating that the 3A-PCL macromer promotes the efficient exfoliation process of graphite. The resulting graphene nanosheets have a substantial reduction in electrical resistance compared to pristine graphite. For example, the 3/10 and 5/10 graphite/3A-PCL composites had electric resistances of the 312 and 348 Ohm, respectively, which were approximately 1.5–2.0 times lower than pristine graphite (544 Ohm) and the 1/10 graphite/3A-PCL composite (665 Ohm). These results further indicate that the combination of surface hydrophilicity and electrical conductivity properties that can be tailored for suitability suggests that these composites have strong potential for electrical stimulation cell culture applications.

### 2.2. Graphite/3A-PCL Composites for Cell Culture Applications

**Figure 3:**
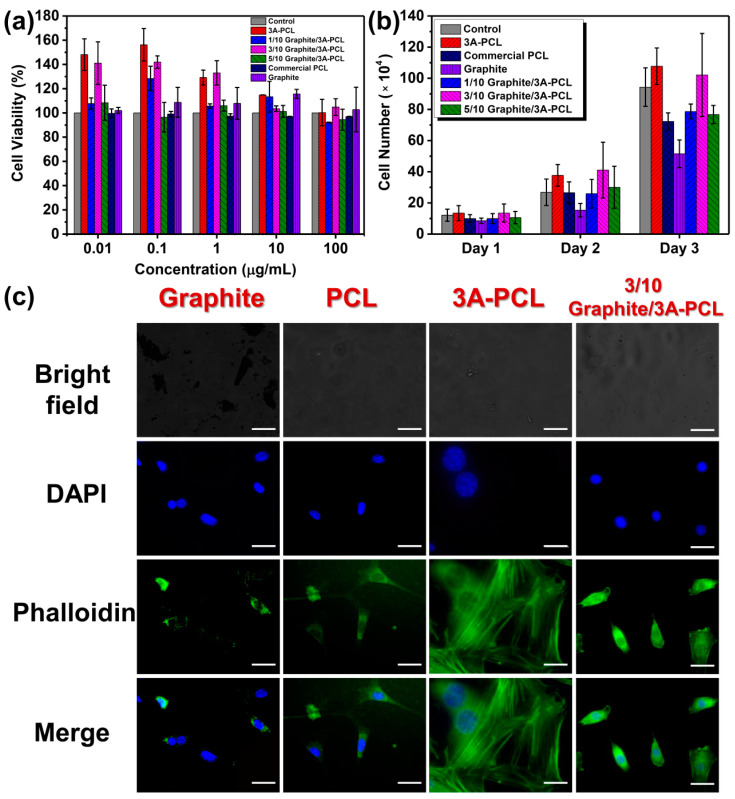
(**a**) The viability of NIH/3T3 cells incubated with pristine graphite, PCL, 3A-PCL, and graphite/3A-PCL composites for 24 h. These viability results were obtained by MTT assay. (**b**) The numbers of NIH/3T3 cells on the graphite, PCL, 3A-PCL, and graphite/3A-PCL substrates after 1–3 days of culture. (**c**) CLSM images of NIH/3T3 cells cultured on pristine graphite, PCL, 3A-PCL, and 3/10 graphite/3A-PCL substrates. After 24 h of culture, the resulting cells were stained with green-fluorescent phalloidin and blue-fluorescent DAPI. White scale bars are 20 µm in all images.

We therefore further explored the structural stability and cytotoxic activity of the graphite/3A-PCL composites toward normal mouse embryonic fibroblasts (NIH/3T3 cells) and sheep red blood cells (SRBCs). As shown in Figure 3a, all sample solutions at concentrations ranging from 0.01 μg/mL to 100 μg/mL showed no significant effect on the viability of NIH/3T3 cells after 24 h treatment using MTT [3-(4,5-dimethylthiazol-2-yl)-2,5-diphenyltetrazolium bromide] assays. Thus, the graphite/3A-PCL composites exerted a low cytotoxic effect on normal NIH/3T3 cells. More surprisingly, the SRBC hemolysis assay clearly demonstrated that the graphite/3A-PCL composites appeared to exhibit greater structural stability and biocompatibility with SRBCs than pristine graphite does (see Supporting Information, Figure S1). This might be attributable to the reversible A-A hydrogen-bonding interactions within the composite matrix (see Figure S2 for the explanation and results), reducing the hemolytic effect of graphite and enabling its potential use for in vivo applications [39,40]. To further evaluate the effects of the hydrogen-bonded adenine moieties and self-assembled structures on the growth of cell numbers, NIH/3T3 cells were seeded on commercial PCL film as a control substrate and on spin-coated 3A-PCL and composite films, before being incubated at 37 °C for various periods (24, 48 and 72 h). After 72 h incubation, the average number of cells in pristine 3A-PCL (1.1 × 10^6^) was 1.5 times higher than on commercial hydrophobic PCL film (7.2 × 10^5^; Figure 3b), indicating that the adenine molecules on the end groups of PCL-enhanced surface hydrophilicity promotes accelerated cell growth in NIH/3T3 cells [19]. Interestingly, the spin-coated 3/10 graphite/3A-PCL substrate was similar in cell growth and number (1.0 × 10^6^) to pristine 3A-PCL, suggesting that the self-assembled structures of 3A-PCL can play a major role in facilitating the proliferation and survival of cells even in the presence of exfoliated graphene nanosheets. However, the 1/10 and 5/10 graphite/3A-PCL substrates had significantly lower cell numbers compared to 3/10 graphite/3A-PCL substrate—cell numbers decreased to approximately 7.7 × 10^5^ in both—suggesting that the exfoliated graphene surfaces are optimally covered by 3A-PCL to achieve a desired surface morphology when the blending ratio of 3A-PCL and graphite is 3:10 [38]. Thus, a further decrease or increase in the 3A-PCL content in the composites apparently alters the surface roughness and wettability in a significant way, leading to a decrease in the total number of produced NIH/3T3 cells. These results confirm that tuning the 3A-PCL content of composites can allow the efficient control of cellular growth and proliferation in adherent cells. This is perhaps due to the presence of strong, multiple high-affinity interactions between NIH/3T3 cells and the hydrogen-bonded adenine group of 3A-PCL, creating a cell culture platform with tailorable physical substrate properties [19].

To confirm the results of cell growth and assess the interaction and relationship at the interface between the adenine units, exfoliated nanosheets and cell surface, confocal laser scanning microscopy (CLSM) was employed to observe the cell cytoskeleton (F-actin, green) and nuclei (bright blue) using phalloidin and 4′,6-diamidino-2-phenylindole (DAPI) staining, respectively. The NIH/3T3 cells seeded and cultured for 24 h on pristine graphite or PCL films displayed an unhealthy cellular shape, insufficient cell density and lacked the characteristic features of the filamentous cytoskeletal network in cell morphology (Figure 3c). Thus, NIH/3T3 cells did not seem to attach to the hydrophobic graphite or PCL surfaces, leading to the inhibition of cell movement and little proliferation. In contrast, NIH/3T3 cells did adhere, spread, and grow well on 3A-PCL film and generated a large area of highly aligned fibroblast-like morphology with the elongation of dendritic filopodia, indicating that the introduction of the adenine moieties in the PCL structure effectively improved the binding affinity between the cell surface and the adenine-modified PCL substrate. This increased binding affinity in turn helped accelerate the cell alignment into clusters from adjacent cells via intercellular adhesions, further promoting cell migration and proliferation. Surprisingly, on the 3/10 graphite/3A-PCL film, NIH/3T3 cells exhibited a highly uniform cellular structure without the presence of a large area covered by filamentous cytoskeletal structures on the substrate. The restricted formation of cytoskeletal filamentous networks between neighboring cells was possibly due to the presence of exfoliated graphene nanosheets within the composites, altering the mechanism of cell proliferation and inducing a change in the cellular behavior or characteristics [41,42]. While 3A-PCL can help maintain the rate of cell growth, these changes in cell behavior led to differential morphological attributes in NIH/3T3 cells when compared to cells cultured in pristine 3A-PCL substrate. Thus, it appears that the combination of exfoliated graphene and bioactive 3A-PCL can create a surface with high cell affinity and tailorable effects on cell culture and that the resulting composite substrate can be efficiently controlled to regulate the cellular functions involved in cell growth, proliferation, and survival. This has the potential to play a vital role in cell and tissue cultures [43].

### 2.3. Evaluation of the In Vitro Wound-Healing Activity on Conductive Bioactive Substrates

**Figure 4:**
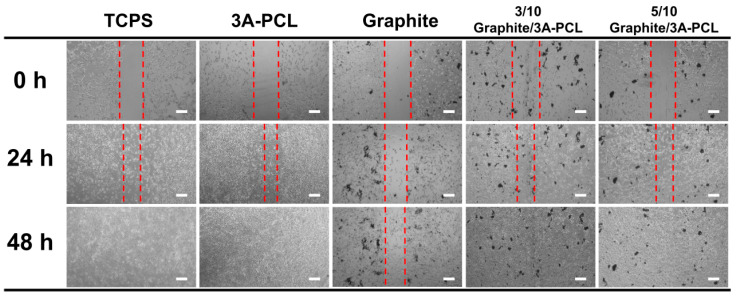
Results of in vitro scratch wound healing assay. Linear scratches were created in the monolayer of NIH/3T3 cells cultured on TCPS, 3A-PCL, graphite, 3/10 and 5/10 graphite/3A-PCL substrates. Images of the scratched areas were monitored through a phase-contrast microscope at 0, 24 and 48 h after wounding. The dashed red lines denote wound edges. All white scale bars represent 100 μm.

**Figure 5:**
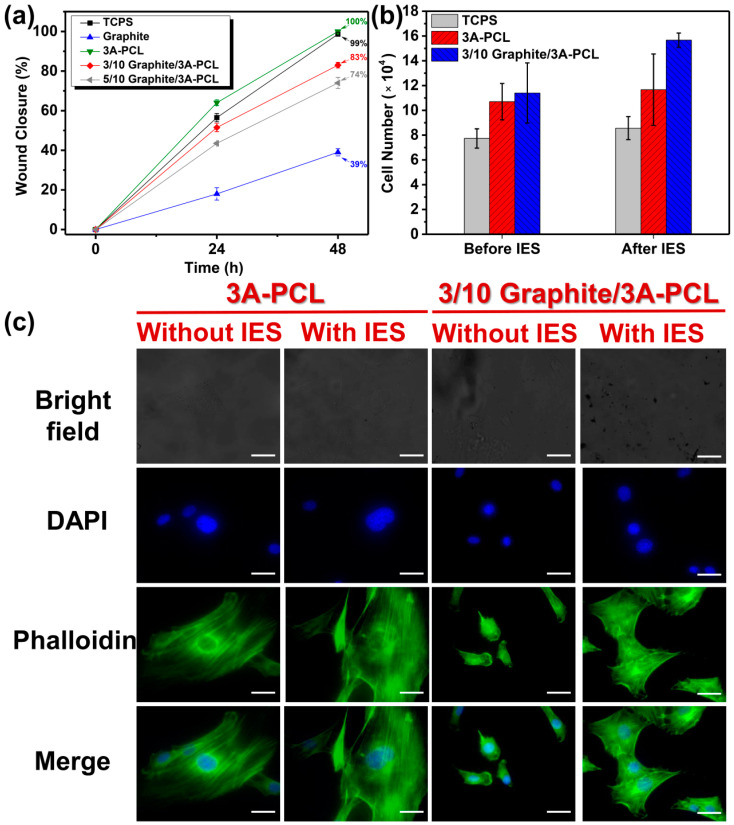
(**a**) The healing rate (percentage) of NIH/3T3 cells by scratch wound healing assay cultured on TCPS, 3A-PCL, 3/10, and 5/10 graphite/3A-PCL substrates at 0, 24, and 48 h after scratching. The (**b**) numbers and (**c**) CLSM images of NIH/3T3 cells harvested on TCPS, 3A-PCL and graphite/3A-PCL substrates treated with or without IES at 0.1 V for 24 h. White scale bars in (**c**) are 20 µm in all images.

To determine whether graphite/3A-PCL composites can significantly improve NIH/3T3 cell growth behavior, we evaluated the effects on cell attachment, migration, and proliferation, using an in vitro scratch wound healing assay [44,45]. As shown in Figure 4 and Figure 5a, after 24 h of culture, the wound closure percentages of NIH/3T3 cells on pristine 3A-PCL can reach up to 64.5 ± 1.4%, whereas cells on control tissue culture polystyrene (TCPS) substrate had only 56.5 ± 2.1% wound closure. We thus conclude that the 3A-PCL substrate can promote cell adhesion and enhance cell spreading and migration through specific interactions between NIH/3T3 cells and hydrophilic adenine-functionalized 3A-PCL substrate, thereby accelerating cell proliferation and wound closure. After 48 h, NIH/3T3 cells on the 3A-PCL and TCPS substrates showed nearly complete wound closure, revealing that 3A-PCL can be used as a high-efficiency bioactive scaffold for cell and tissue culture applications. In contrast to pristine 3A-PCL, graphite substrates led to a delay in wound closure efficiency, with the scratch-damaged NIH/3T3 monolayer showing only 38.5 ± 2.0% wound closure after 48 h. This suggests that the hydrophobicity of the graphite surface suppresses cell growth, migration, and cycle progression. However, with the incorporation of 3A-PCL into the graphite matrix, the resulting composite substrates significantly enhance the wound closure rate in NIH/3T3 cells. For example, the wound closure efficiency of NIH/3T3 cells on the 3/10 and 5/10 graphite/3A-PCL substrates after 48 h of culture reached 83 ± 1.4% and 74 ± 2.8%, respectively. Overall, these findings demonstrate that adjusting the content of 3A-PCL is critical to controlling the physical properties of exfoliated graphene nanosheets [38] and the resulting composites can be used as a bioactive substrate to efficiently manipulate and regulate the cell growth and wound healing ability, eventually achieving desirable cell performance in cell culture.

### 2.4. Assessment of Cell Growth on Conductive Bioactive Substrates with IES

Given that graphite/3A-PCL composites have excellent electrical conductivity, we decided to further explore whether these newly developed substrates could enhance cell growth and manipulate the cell morphology and shape through the use of an indirect-current electric field [46,47,48]. The cell culture experiment using IES was performed under low electric voltage at 0.1 V/mm (approximately 1~2 mA current intensity); this level does not affect cell growth or have negative effects during cell culture [49,50]. After 24 h of culture under IES, the number of NIH/3T3 cells on the 3:10 graphite/3A-PCL substrate increased from 11.4 × 10^4^ to 15.7 × 10^4^ while little change in cell number was seen on control TCPS or pristine 3A-PCL substrate with IES treatment (Figure 5b). These results suggest that exfoliated graphite nanosheets present in composites create an electric field-sensing medium that effectively stimulates the structural motion of the composite under low-voltage electric fields. This probably promotes an increased interaction of composite and cells at the interface, leading to a significant increase in total cell number. To explore how the graphite/3A-PCL substrates under IES treatment influence cell morphology, NIH/3T3 cells were cultured with pristine 3A-PCL or graphite/3A-PCL substrates for 24 h under 0.1 V, and then observed under CLSM to detect changes in cell shape and morphology. The CLSM images showed no significant differences in cell morphology on the pristine 3A-PCL substrate before and after IES treatments (Figure 5c). In contrast, cells on the 3/10 graphite/3A-PCL substrate cell morphology changed remarkably after IES treatment, with cells displaying interlocking and close-packing bundles of spindle-shaped cells of increased overall cell area and pseudopod numbers after IES treatment (Figure 5c, far right). The increased coalescence and proliferation of cells with neighboring cells can be attributed to the fact that exfoliated graphene nanosheets in graphite/3A-PCL composites not only act as an electrical stimulation unit, like an “active trigger”, to facilitate the segmental motion of the 3A-PCL polymer chains, but also to efficiently facilitate interaction between the cells and substrate through the effect of an external electric field. This facilitated interaction accelerates the formation of a closely connected cell morphology via the promotion of intercellular adhesion, resulting in a significant increase in total cell numbers on the substrate. Overall, we concluded that the introduction of the adenine moieties in the PCL matrix substantially enhances the binding affinity with cells, which promotes the attachment of cells to the substrate and regulates cellular characteristics, i.e., adhesion, proliferation, and migration. Incorporating graphite into the 3A-PCL substrates enabled the effective tuning of the wettability and conductivity of the substrate surface and thus altered the growth behavior and characteristics of the cells. Importantly, under an indirect-current electric field of only 0.1 V, the graphite/3A-PCL substrates rapidly stimulated the spread and proliferation of cells and significantly increased the total cell numbers. Thus, the adenine and exfoliated graphene-containing bioactive substrates exhibit unique physical and biological properties that efficiently enhance cell growth and manipulate cell morphology and function through IES-responsive characteristics. These important features suggest great potential for a wide variety of biomedical applications, especially as a highly effective scaffold for tissue and cell cultures [43].

## 3. Materials and Methods

Details regarding the synthetic procedures, the cell experiments and instrumentation used in this study are given in the Supplementary Information.

## 4. Conclusions

In summary, we successfully created a high-performance conductive supramolecular nanocomposite containing a hydrogen-bonded adenine-functionalized PCL and exfoliated graphene nanosheets that serve as a highly efficient bioactive substrate for the cell culture and manipulation of cell biophysical properties. The exfoliation of graphite within the 3A-PCL matrix promotes the formation of well-dispersed graphene nanosheets with unique structural and physical properties due to the presence of strong interaction between the exfoliated graphene nanosheets and the self-assembled nanostructures of 3A-PCL. The resulting spin-coated composite films can be easily tuned by altering the blending ratio of the graphite and 3A-PCL to obtain the required level of surface roughness and achieve the desired surface wettability and electrical conductivity. The combination of a wide range of tunable physical properties and stable thermo-reversible behavior of graphite/3A-PCL composites is rare and has strong potential for use as cell culture substrates or tissue culture scaffolds. When these newly developed composites were evaluated under in vitro environmental conditions, they exhibited extremely low cytotoxic activity against NIH/3T3 normal cells, high structural stability, and biocompatibility in the SRBC-containing medium. Cell culture, scratch experiments, and fluorescence images confirmed that pristine 3A-PCL substrates can efficiently interact with cell surfaces to enhance cell attachment, spreading, migration, and proliferation. With the incorporation of graphite into the 3A-PCL matrix, the resulting composite substrates can efficiently regulate cellular functions involved in cellular morphological features, without affecting wound healing abilities. More importantly, placing the graphite/3A-PCL substrates under an indirect current electric field of only 0.1 V rapidly stimulated cell responses in terms of adhesion, spreading, viability, and proliferation, leading to a substantial increase in the total cell numbers and a significant alteration in cell morphology, especially a gradual increase in cell size distribution. The presence of both adenine moieties and exfoliated graphene nanosheets within the composite substrates are crucial for the manipulation of cell growth, morphology, and functions by IES-responsive characteristics. This newly created strategy provides a simple, rapid, and efficient path to produce biocompatible and biodegradable conductive supramolecular nanocomposites for the development of multifunctional bioactive substrates that can substantially improve the cell culturing process.

## Data Availability

Not applicable.

## Supplementary Materials

The following supporting information can be downloaded at: https://www.mdpi.com/article/10.3390/ijms23084332/s1.
