# Supplementary material for: Conductive Supramolecular Polymer Nanocomposites with Tunable Properties to Manipulate Cell Growth and Functions
Source: Int J Mol Sci. 2022 Apr 14;23(8):4332. doi: 10.3390/ijms23084332 (PMC9032009; doi:10.3390/ijms23084332)
Supplement: Supplementary file 1 [file ijms-23-04332-s001.zip › Revised Suppoting Information_CC_.pdf]

Suppoting Information

Conductive Supramolecular Polymer Nanocomposites with Tunable Properties to Manipulate Cell Growth and Functions

Cheng-You Wu,[1] *Ashenafi Zeleke Melaku,*[1] *Fasih Bintang Ilhami,*[1] *Chih-Wei Chiu*[2] *and Chih-Chia Cheng*[1,3]*

1. Graduate Institute of Applied Science and Technology, National Taiwan University of Science and Technology, Taipei 10607, Taiwan. E-mail: cccheng@mail.ntust.edu.tw
2. Department of Materials Science and Engineering, National Taiwan University of Science and Technology, Taipei 10607, Taiwan.
3. Advanced Membrane Materials Research Center, National Taiwan University of Science and Technology, Taipei 10607, Taiwan.

Experimental section

Materials

All chemicals, including graphite, adenine, polycaprolactone triol (average molecular weight (M_n) ~900 g/mol), polycaprolactone (PCL; M_n = 80,000 g/mol), acryloyl chloride, potassium *tert*-butoxide , tetrahydrofuran (THF), dimethylformamide (DMF) and chloroform were purchased from Sigma-Aldrich (St. Louis, MO, USA) at the highest analytical grade available.

NIH/3T3 (mouse embryonic fibroblast) cell lines were purchased from ATCC (American Type Culture Collection, Manassas, VA, USA). DMEM (Dulbecco's Modified Eagle's Medium), MTT [3-(4,5-dimethylthiazol-2-yl)-2,5-diphenyltetrazolium bromide], penicillin, streptomycin, PBS (phosphate buffered saline), FBS (fetal bovine serum), phalloidin and DAPI (4,6-diamidino-2-phenylindole) were purchased from Invitrogen Corporation (Carlsbad, CA, USA).

Composite Synthesis

Synthesis of three-arm adenine end-capped polycaprolactone (3A-PCL): 3A-PCL was obtained using the Michael addition reaction of adenine and PCL triacrylate in DMF at 70 °C for 48 h; details of the preparation procedure are given in our previous work [S1].

Production of graphite/3A-PCL composites: Graphene containing biodegradable polymer composite was prepared by mixing 1-5 mg of graphite directly with 10 mg of 3A-PCL (blend ratio from 1:10 to 5:10) in THF and then exfoliated via ultra-sonication treatment for 3 h. Following exfoliation, three-quarters of the upper solution was removed and then the remaining solvent was removed using a rotary evaporator. Once the product was dried, it was kept in a dry box for further use [S1].

Physical Properties of Composites

Variable temperature Fourier Transform Infrared Spectroscopy (VT-FTIR): We obtained Fourier transform infrared (FTIR) data using an FTIR spectrometer (PerkinElmer Spectrum Two FTIR, Buckinghamshire, UK) at different temperatures over several scans with a resolution of 2.0 cm^{-1}. Variable temperature measurements were taken over the wide temperature range of 40-80 °C at a steady heating rate of 1.0 °C/min under ambient atmospheric conditions.

Water Contact Angle (WCA) Measurements: To assess wettability of the composite, we did a WCA experiment with a contact angle meter (VCA-2500XE AST Products, VCA Optima, Billerica, MA, USA) at 25 °C. A high-resolution digital charge-coupled device camera was used to capture the WCA images when a water drop was deposited on sample coated silica wafer; built-in software calculated the WCA formed between the droplet and solid substrate.

Conductivity measurement: The electrical conductivity behavior of graphene containing 3A-PCL was evaluated using a four-probe electronic device (RT-80, Napson, Tokyo, Japan). Samples spin coated onto silica welfare substrate at 1500 rpm for 15 s were dried in a vacuum oven at 30 °C for one day and electrical resistivity was directly measured under low humidity level (approximately 35%) at room temperature. The corresponding resistance of the surface of substrates was calculated using the sheet resistance equation as described in our previous work [S1].

Atomic force microscopy (AFM): The number of layers of graphene nanosheets obtained from graphite/3A-PCL composites was confirmed with AFM (NX10, AFM Park Systems, Suwon, South Korea) in open environment at room temperature. Exfoliated graphene solution was spin-coated on silica wafer substrate at 1500 rpm for 15 s and dried in a vacuum oven for 24 h at 25 °C and then taken for AFM measurement. Finally, the layer thickness of obtained graphene nanosheets was determined using XEI software (PARK Systems Inc.).

Cell Culturing with Composites

Cell Culture: NIH/3T3 cells was cultured in a cell incubator, with DMEM (90%) containing FBS (10%) and penicillin/streptomycin (1%), with 5% CO_2 at 37 °C. NIH/3T3 cells were collected with trypsin and resuspended in cold PBS. Then, viable cells were stained with 0.1% trypan blue and counted under the microscope before use in experiments.

Cytotoxicity Evaluations: The cytotoxicity of PCL, pristine graphite, 3A-PCL and graphite/3A-PCL composites against fibroblast NIH/3T3 cells was estimated with the MTT assay method. First, when the cells reached a state of encounter of approximately 80%–90%, they were cultured into a 96-well plate with density of 1×10^5 cells/well for 24 h. After 24 h, the medium was removed and the cells were washed with PBS, subsequently added samples containing DMEM medium at various concentrations and incubated for additional 24 h. We next removed the medium and replaced it with the MTT assay, incubating the cells for 4 h. After 4 h, the MTT solution was removed and we added 100 µl of DMSO. The plate was then analyzed by an ELISA microplate reader using 570 nm (ELx808i, BioTek. Instruments, USA) and cell viability percentage was estimated with the equation:

$$Cell\ Viability = \frac{Absorbance\ of\ treated\ cell}{Absorbance\ of\ control\ cell}\ x\ 100.$$

Assessment of Cell Growth on Supramolecular Composite Substrates with Indirect Electrical Stimulation (IES): First, all samples were coated onto glass substrate and sterilized. Then, we placed sample-coated glass substrate in 12-well plates and seeded fibroblast NIH/3T3 cells (1×10^4 cells) directly onto sample-coated glass substrate. Next, the top and the bottom of the 12-well plates were covered by 2 mm-thick indium tin oxide (ITO) conductive glass (sizes, 8 × 8 mm), treated with/without IES at 0.1 V in an incubator and cultured at 37 °C for various time (24, 48 or 72 h). The cells on the sample-coated substrates were viewed using an optical microscope under bright-field transmitted light after being rinsed twice with media. The number of cells in each substrate was meticulously counted for a

selected area using an inverted light microscope (Nikon Eclipse TE2000, Nikon, Tokyo, Japan) at different magnifications.

Confocal Laser Scanning Microscopy (CLSM): NIH/3T3 cells were cultured at 37 °C on sample-coated substrates with or without IES at 0.1 V for 24 h. Sample-coated substrates were then washed with cold PBS and fixed in 4% paraformaldehyde solution for 15 min. After triple-washing with cold PBS, cells were stained with phalloidin for 10 min and DAPI for 10 min. Finally, we visualized samples using a confocal microscope (iRiS™ Digital Cell Imaging System, Logos Biosystems, South Korea).

Evaluation of Wound Healing on Supramolecular Composite Substrates and Hemolytic Activity: First, NIH/3T3 cells were seeded at 1×10^4 cells onto sample-coated substrates and cultured for 24 h. Then, a sterile 1.0 mL pipette tip was used to create a mechanical scratch wound on the confluent NIH/3T3 monolayer culture, making a cell-free region. Cells were cultured for an additional 48 h and observed for healing at 24 and 48 h. The wound healing process of cells was observed using an optical microscope under bright-field transmitted light and calculated using ImageJ software:

$$\text{Wound Closure \%} = \left[\frac{A_{t=0h} - A_{t=\Delta h}}{A_{t=0h}}\right] \times 100\%,$$

where, $A_{t=0h}$ = the area of wound measured immediately after scratched (t = 0 h) and $A_{t=\Delta h}$ = the area of wound measured 24 or 48 h after scratched.

Sheep red blood cells (SRBCs, Cosmo Bio, Tokyo, Japan) were used to analyze hemolytic activity of sample and evaluated as previously described [S2].

Statistical Analysis

All results are provided as means with standard deviations from at least three replicate trials.

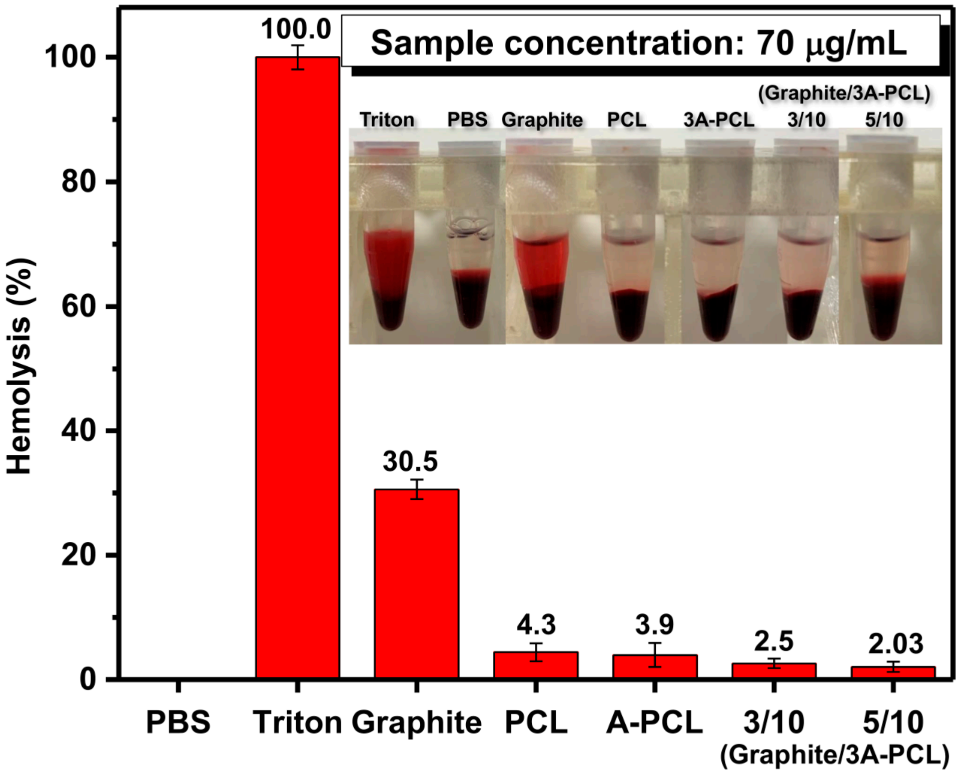

Figure S1: Photographs and SRBC hemolysis results after incubation with 70 μg/mL of graphite, PCL, 3A-PCL and graphite/3A-PCL composites.

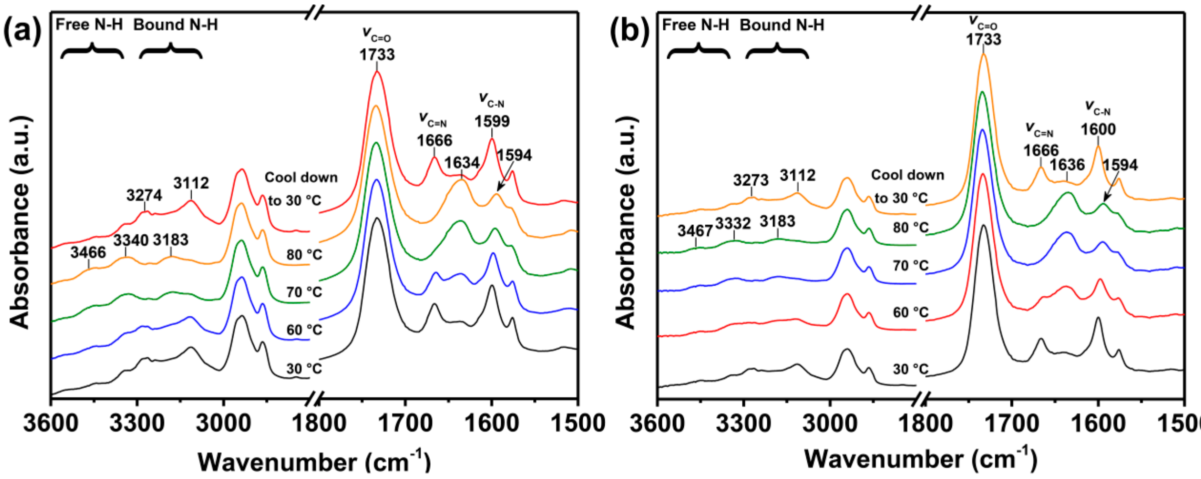

Figure S2: VT-FTIR spectra of **(a)** 3A-PCL and **(b)** 3/10 graphite/3A-PCL composites at various temperatures (30−80 °C) and after cooling down to 30 °C.

Variable-temperature Fourier transform infrared (VT-FTIR) measurement was carried out to elucidate SRBC hemolysis results (Figure S1) and investigate the reversible hydrogen-bonding behavior of 3A-PCL and its composites. As shown in Figure S2a, the VT-FTIR spectra of 3A-PCL at 30 °C exhibited two N-H characteristic peaks at 3112 cm^{-1} and 3274 cm^{-1}, which indicate self-complementary hydrogen-bond formation between the adenine moieties of 3A-PCL [S3]. Other peaks in the low wavenumber region were observed at 1733 cm^{-1}, 1666 cm^{-1} and 1599 cm^{-1}, which correspond to carbonyl-stretching vibrations of amorphous esters and strongly hydrogen-bonded C-N/C=N stretching vibrations of adenine moieties, respectively [S4]. When the environmental temperature gradually increased from 30 °C to 80 °C, the hydrogen-bonded N-H peaks at 3112 cm^{-1} and 3274 cm^{-1} shifted significantly to higher wavenumber and the stretching vibration of a free N-H peak was clearly observed at 3466 cm^{-1}, while the hydrogen-bonded C-N/C=N peaks at 1666 cm^{-1} and 1599 cm^{-1} shifted to lower wavelength, indicating that an elevated temperature results in a gradual destruction of the A-A hydrogen-bonding interactions within the 3A-PCL matrix [S5]. However, when the environmental temperature was slowly cooled from 80 °C to 30 °C, the FTIR spectrum returned completely to its original state, revealing that the adenine moieties of 3A-PCL

display a stably thermoreversible self-complementary A-A hydrogen-bonding interaction (Figure S2a).

The VT-FTIR spectra of the 3/10 graphite/3A-PCL composites in Figure S2b showed same trends as that of pristine 3A-PCL, further confirming that the presence of exfoliated graphene nanosheets within the composites did not affect the hydrogen-bond behavior of 3A-PCL, which maintained their thermoreversible nature. These results might reflect the formation of high-aspect-ratio exfoliated graphene nanosheets and high affinity binding between the graphene surface and 3A-PCL that improve the thermoreversible stability of the resulting composites [S1]. In addition, these observations compliment the results of the SRBC hemolysis assay, i.e., show that 3A-PCL adsorbed on the surface of exfoliated graphene nanosheets could substantially improve the biocompatibility of the graphene with SRBCs (Figure S1).

References:

S1. Wu, C. Y.; Melaku, A. Z.; Chuang, W. T.; Cheng, C. C. Manipulating the self-assembly behavior of graphene nanosheets via adenine-functionalized biodegradable polymers. *Appl. Surf. Sci.* **2022**, *572*, 151437.

S2. Cheng, C. C.; Yang, X. J.; Fan, W. L.; Lee, A. W.; Lai, J. Y. Cytosine-functionalized supramolecular polymer-mediated cellular behavior and wound healing. *Biomacromolecules* **2020**, *21*, 3857–3866.

S3. Muhabie, A. A.; Cheng, C. C.; Huang, J. J.; Liao, Z. S.; Huang, S. Y.; Chiu, C. W.; Lee, D. J. Non-covalently functionalized boron nitride mediated by a highly self-assembled supramolecular polymer. *Chem. Mater.* **2017**, *29*, 8513–8520.

S4. Hayashi, M.; Tournilhac, F. Thermal stability enhancement of hydrogen bonded semicrystalline thermoplastics achieved by combination of aramide chemistry and supramolecular chemistry. *Polym. Chem.* **2017**, *8*, 461–471.

S5. Cheng, C. C.; Chang, F. C.; Chen, J. K.; Wang, T. Y.; Lee, D. J. High-efficiency self-healing materials based on supramolecular polymer networks. *RSC Adv.* **2015**, *5*, 101148–101154.
